# Transhepatic embolization of a pancreatico-duodenal arteriovenous malformation: Application of retrograde pressure cooker technique with ethylene-vinyl alcohol copolymer

**DOI:** 10.1016/j.radcr.2026.02.051

**Published:** 2026-03-20

**Authors:** Philip Nguyen Powanda, Elizabeth A. Rowland, Kevin Liu, Kenneth S. Zurcher

**Affiliations:** a University of Arizona College of Medicine–Phoenix, Phoenix, AZ, USA; b Division of Gastroenterology and Hepatology, Banner University Medical Center–Phoenix, Phoenix, AZ, USA; c Division of Interventional Radiology, Banner University Medical Center–Phoenix, Phoenix, AZ, USA

**Keywords:** Pancreatic arteriovenous malformation, Transhepatic embolization, Pressure cooker technique, Embolization

## Abstract

Pancreatic arteriovenous malformations are rare vascular anomalies that may present with gastrointestinal bleeding, abdominal pain, or portal hypertension. Their management is challenging, with surgical resection carrying a significant risk of morbidity. Endovascular embolization offers a less invasive alternative; however, success is often limited in cases with multiple arterial feeders. We present a 65-year-old woman with cirrhosis and upper gastrointestinal hemorrhage from a Yakes type III pancreatic arteriovenous malformation. Given her poor surgical candidacy and complex arterial inflow, transvenous retrograde embolization via a transhepatic approach was performed using a modified pressure-cooker technique with coils and Onyx. Complete nidus obliteration was achieved following staged interventions. This represents the first reported successful use of a retrograde pressure-cooker technique with Onyx in a pancreatic arteriovenous malformation.

## Introduction

Pancreatic arteriovenous malformations (PAVMs) are rare vascular anomalies involving abnormal connections between pancreatic arteries and veins and represent approximately 0.9% of all gastrointestinal AVMs [1]. They are primarily congenital and often associated with hereditary hemorrhagic telangiectasia (HHT), though may also arise from trauma, pancreatitis, or malignancy. While often asymptomatic, PAVMs can lead to gastrointestinal bleeding, abdominal pain, pancreatitis, and portal hypertension.

Given the low incidence of symptomatic PAVMs, no established guidelines exist for their management. Current strategies include endovascular embolization or surgical resection, including pancreaticoduodenectomy, a potentially morbid operation. Complications can include bleeding related to complex vascular supply and anatomy, infection, or diabetes [[2], [3], [4]]. When feasible, endovascular embolization may offer an effective, less invasive alternative.

This report describes a 65-year-old woman with PAVM complicated by upper gastrointestinal hemorrhage who underwent embolization using a novel application of the pressure-cooker technique (PCT) through a retrograde transhepatic portal approach.

## Case report

A 65-year-old woman with complex medical history including fibromyositis, fibromyalgia, and compensated cirrhosis secondary to hepatitis C presented with 1 week of melena and epigastric pain. She was hypotensive (BP 90/50 mmHg) and hypoxic on admission, with severe anemia (Hgb 4.3 g/dL), leukocytosis (20.4 K/µL), and lactic acidosis (6.1 mmol/L).

## Imaging

**Fig. 1:**
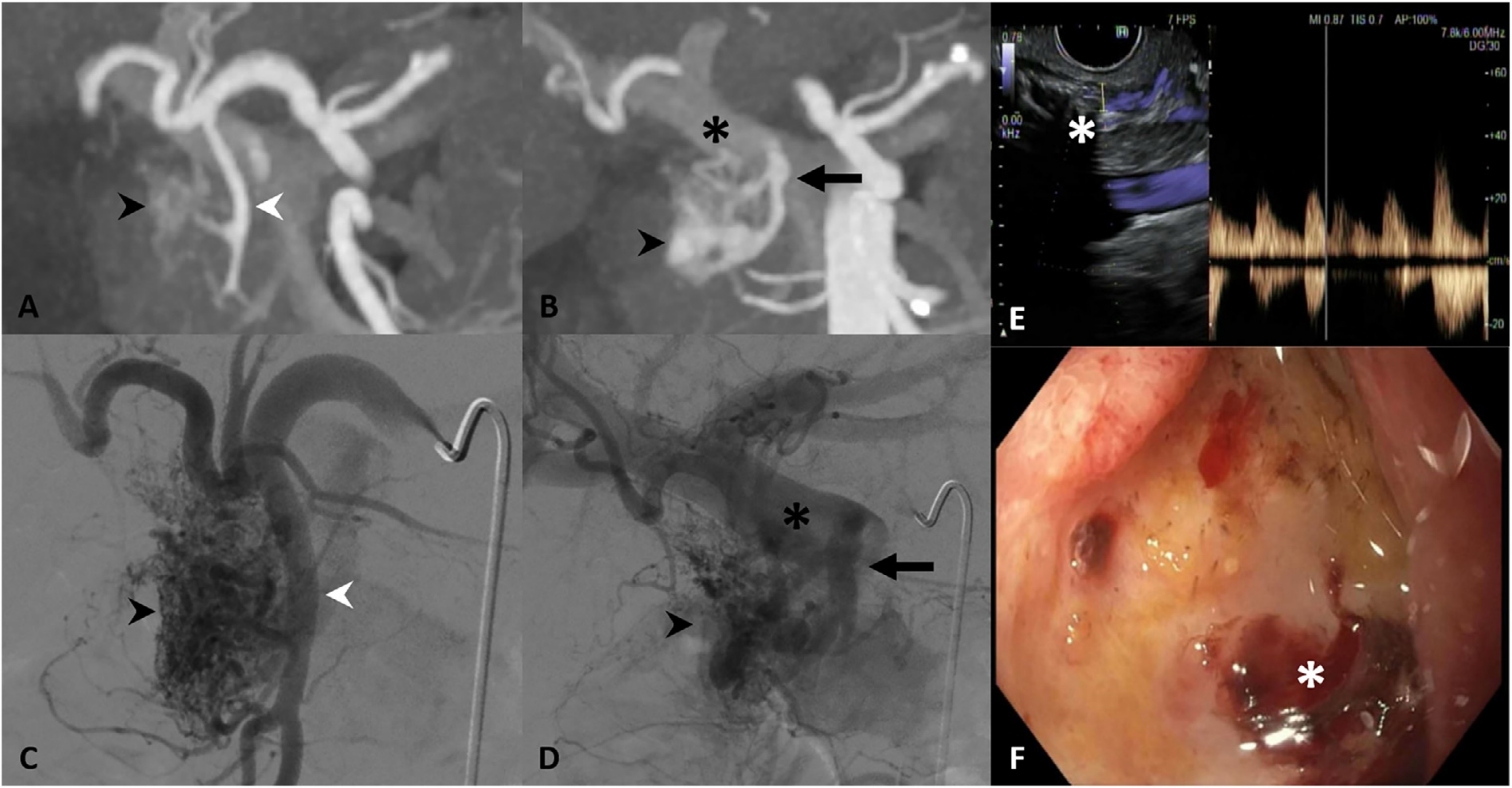
CT, angiographic, and endoscopic depiction of pancreatic arteriovenous malformation (PAVM). (A) Coronal CTA demonstrating complex AVM nidus in the pancreatic head/proximal duodenum (black arrowhead) with arterial inflow from gastroduodenal artery (GDA) branches (white arrowhead), and (B) early venous outflow to the main portal vein (asterisk) via pancreatico-duodenal vein (PDV) (black arrow). (C and D) Corresponding digital subtraction angiograms (DSA) demonstrating multiple GDA inflow vessels and portal outflow. (E) Endoscopic ultrasound (EUS) image demonstrating abnormal vascularity/PAVM in the pancreatic head (white asterisk). (F) Esophagogastroduodenoscopy (EGD) with bleeding ulcer in the proximal duodenum.

A contrast-enhanced multiphasic CT angiogram revealed a hypervascular nidus in the pancreatic head/duodenal wall, with inflow from gastroduodenal artery (GDA) branches and outflow via a portal vein branch, suggestive of PAVM (Fig. 1). Esophagogastroduodenoscopy (EGD) showed a bleeding ulcer in the first/second portion of the duodenum, concordant with CT findings. Given the patient’s cirrhosis and poor surgical candidacy, a complex transarterial/transportal embolization was planned.

## Intervention

Celiac, hepatic, and GDA angiography demonstrated a large, complex PAVM involving the pancreatic head and proximal duodenal wall, with multiple arterial feeding vessels arising from the GDA, pancreatico-duodenal artery (PDA), supra-duodenal artery, and proper hepatic artery (Fig. 1). Delayed phase imaging showed brisk abnormal venous drainage into the portal vein via a single pancreatico-duodenal vein (PDV). Due to numerous inflow branches, transarterial embolization was felt unlikely to achieve complete embolization; given a single outflow vein (Yakes type III), transvenous retrograde embolization was pursued.

**Fig. 2:**
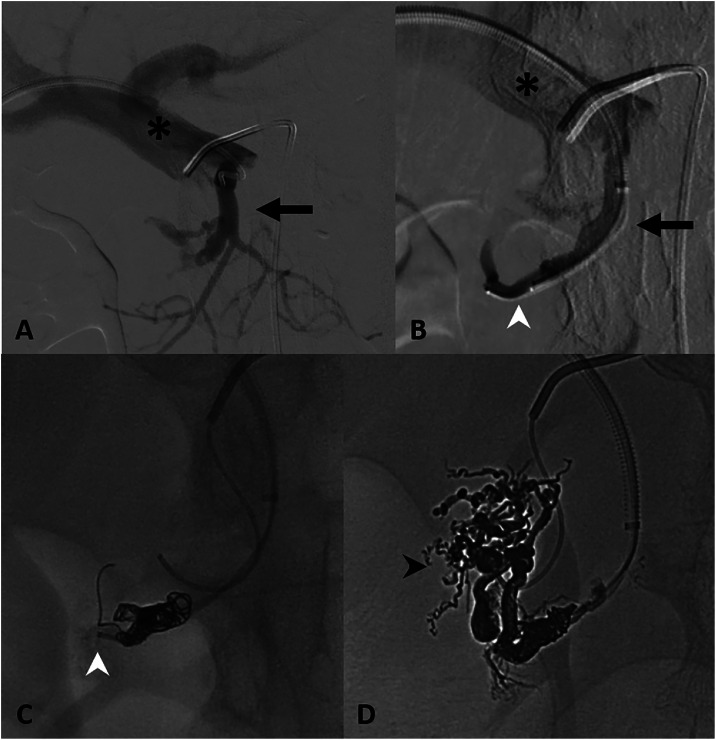
Transhepatic retrograde pressure-cooker embolization. (A) Portal venogram via transhepatic (black asterisk) access, with reverse-curve catheter positioned in the pancreatico-duodenal vein (PDV) outflow vessel (black arrow). (B) Selective PDV (black arrow) venogram via staggered dual microcatheters (white asterisk). (C) Pressure-cooker technique demonstrating proximal microcatheter coiling, with distal positioned microcatheter initiating Onyx embolization (white arrowhead). (D) Completion spot image demonstrating retrograde pressure-directed Onyx embolization with casting of the pancreatic arteriovenous malformation (PAVM) nidus (black arrowhead).

Percutaneous transhepatic access of the right portal vein was obtained with a 6 French × 45 cm curved sheath. Utilizing a 5 French reverse-curve catheter, the PDV was selected, and the sheath was advanced into the PDV (Fig. 2). The catheter was exchanged for two 2.4-French microcatheters which were both positioned into the PDV in staggered fashion. Utilizing a modified PCT, 1 microcatheter was used to “jail” the other, with deployment of 5-6 mm detachable Concerto coils (Medtronic, Minneapolis, MN) distal to the second microcatheter positioned more centrally within the nidus–as depicted in Figures 2B and C. Via the proximal microcatheter within the nidus, ethylene-vinyl alcohol copolymer (Onyx 34, Medtronic, Ireland, Dublin) was delivered until plug formation occurred around the coil pack, allowing a 2-minute dwell time. Further embolic was delivered in pressure-directed fashion with the coil pack facilitating retrograde embolization into the nidus and preventing non-target portal embolization. Intermittent 2-minute pauses were performed to facilitate continued embolization of the nidus. Due to persistent vascularity of the nidus with outflow from a superior PDV branch, a second transvenous embolization using the same PCT was performed targeting this branch. In total approximately 2 mL of Onyx 34 was administered.

A final angiogram of the GDA demonstrated no definitive residual nidus opacification or venous drainage. While multiple arterial inflow branches were present as discussed, a single dominant PDA branch previously seen to exclusively supply the nidus was noted. To further reduce theoretical residual arterial inflow, or nidus recurrence, the dominant PDA feeder was embolized with 3 mm detachable coils and a shear-thinning gel embolic. A final angiogram of the GDA demonstrated no residual nidus opacification or venous drainage.

The patient tolerated the procedure well with no immediate complications. The theoretical risk of duodenal ulceration or pancreatitis was considered by operators but mitigated by a predominantly transvenous embolization. This was in distinction to considering an extensive arterial intervention in the gastrointestinal tract with liquid embolics commonly used in AVM treatment (Onyx, ethanol). Consequently, serial lipases were obtained post procedurally—with minimal elevation over 24 hours up to 84 U/L, with near normalization by post procedure day 2 (68 U/L) (reference range 16-63). She remained asymptomatic with no abdominal pain or nausea and was subsequently discharged on post procedure day 2.

## Follow-up and outcome

At 1-month follow up catheter angiography, minimal residual AVM perfusion was identified with new drainage into a superior mesenteric vein branch. As the patient remained asymptomatic, no intervention was performed, and outpatient EGD was planned.

**Fig. 3:**
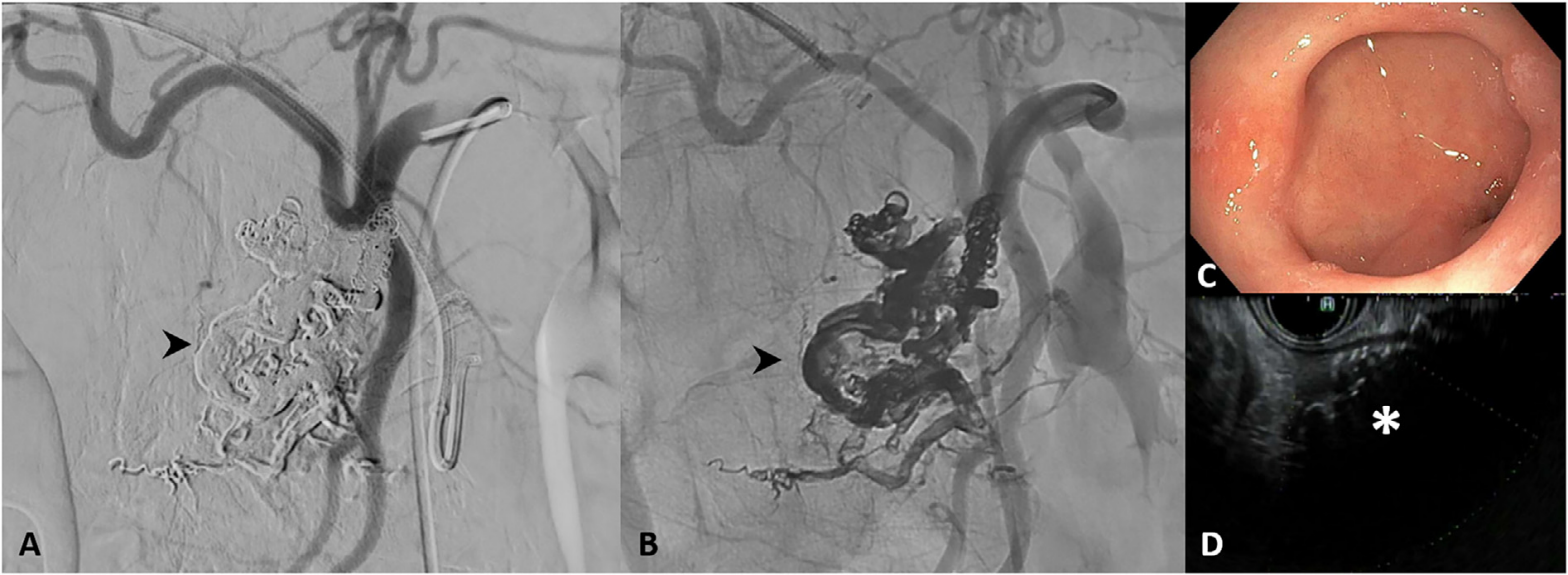
Angiographic and endoscopic images of pancreatic arteriovenous malformation (PAVM) post embolization. (A and B) Common hepatic artery DSA following second embolization procedure demonstrating complete casting of the nidus (black arrowhead) with no residual perfusion of the nidus. (C and D) Follow up esophagogastroduodenoscopy (EGD) demonstrates resolution of proximal duodenal ulcer, with endoscopic ultrasound (EUS) images demonstrating dirty shadowing of the pancreatic head AVM with no residual abnormal vascularity (white asterisk).

At 2-month follow-up, she re-presented with epigastric pain. Endoscopic ultrasound (EUS) and EGD confirmed persistent duodenal ulcer and dilated vessels suggestive of residual AVM. A second transhepatic retrograde pressure-directed embolization with Onyx was performed from the superior mesenteric vein outflow branch, achieving complete nidus obliteration (Fig. 3).

At 3-month follow-up, the patient remained asymptomatic. Repeat EGD/endoscopic ultrasound showed complete resolution of the duodenal ulcer, with no evidence of residual vascularity (Fig. 3). A genetic workup for hereditary hemorrhagic telangiectasia was negative.

## Discussion

While surgical resection remains the most common treatment option for treating PAVMs (approximately 44% in a review of case reports/series by Onozawa et al.), it carries significant risk of complication, particularly in cirrhotic or coagulopathic patients [1,5]. Additional risks include pancreatic insufficiency and postoperative diabetes [6]. Percutaneous embolization or radiotherapy may help preserve pancreatic function while also reducing the risk of surgical complications. Relatively few cases (approximately 40) of PAVM embolization exist in the literature. Most however, are almost entirely composed of antegrade transarterial embolization, with a reported 57.7% success rate [1,7].

Effective transarterial embolization of a PAVM may be challenging if multiple inflow arteries are present; a transvenous pressure-directed or PCT may be an effective alternative in such cases if only 1-2 draining veins are present (Yakes type III). To date only 3 transvenous PAVM interventions have been reported, with only 1 utilizing PCT, presented in a 7-patient case series by Marcelin et al. [1,8]. In this case, an Amplatzer plug (St. Jude Medical, Plymouth, Minnesota) was deployed via transhepatic access in a draining gastroduodenal vein, with a secondary microcatheter used for retrograde embolization/PCT of the nidus with sodium tetradecyl sulfate (STS) and lipiodol. A second transvenous case was presented in this series, in which a pancreatic tail AVM possessed single venous drainage into the splenic vein. Via transsplenic access, a splenic vein stent graft was used to cover the venous outflow of the nidus, followed by direct percutaneous puncture and embolization of the nidus with Onyx and coils. Subsequent splenic vein thrombosis occurred requiring thrombectomy; however, AVM treatment was successful. In a 2021 case report by Onishi et al., a pancreatic head AVM with outflow to the portal and splenic veins was identified [9]. Given presence of ascites, transvenous percutaneous access was obtained via recanalized paraumbilical vein—facilitating access of the portal system and AVM outflow. Direct catheter-based coil embolization was performed in the aneurysmal nidus with successful embolization confirmed via concurrent celiac angiogram.

The PCT was first described by Chapot et al. for cerebral AVMs and was developed to minimize the risk of non-target embolization and allow greater volumes of Onyx to be delivered into the nidus [10]. Although the PCT was initially intended to treat AVMs using an antegrade arterial approach, we demonstrate a successful transvenous adaptation of the neurointerventional technique to the visceral vasculature. To our knowledge, this represents the only successful treatment of a Yakes Type III PAVM via retrograde transvenous PCT administration of Onyx.

## Conclusion

This case highlights the successful utilization of a retrograde pressure-directed/PCT with ethylene-vinyl alcohol copolymer in the embolization of a complex PAVM. In PAVM with multiple arterial feeders, retrograde treatment with a PCT may represent a viable first-line treatment option—particularly in poor surgical candidates.

## Patient consent

Written informed consent for publication was obtained from the patient and can be provided at any time if necessary.
